# Pancreatic stem cells originate during the pancreatic progenitor developmental stage

**DOI:** 10.3389/fcell.2025.1521411

**Published:** 2025-02-18

**Authors:** Krystal Jacques, Brenda L. K. Coles, Derek van der Kooy

**Affiliations:** ^1^ Donnelly Centre for Cellular & Biomolecular Research, University of Toronto, Toronto, ON, Canada; ^2^ Institute of Medical Science, University of Toronto, Toronto, ON, Canada; ^3^ Department of Molecular Genetics, University of Toronto, Toronto, ON, Canada

**Keywords:** pancreatic progenitor, pancreatic development, clonal assays, human ES cell differentiation, mouse primary cell culture, beta cell (β cell), pancreatic stem cell

## Abstract

Previously isolated adult pancreatic precursors called pancreatic multipotent progenitors (which make both pancreatic endocrine and exocrine cell types) originate from the Pancreatic Duodenal Homeobox 1 (PDX1) pancreatic developmental lineage. The embryonic time point at which adult pancreatic multipotent progenitor cells emerge has not been established. We have employed the use of two models: a human embryonic stem cell (hESC) to beta-cell cytokine-induced differentiation protocol and a mouse lineage tracing model during early development to isolate clonal pancreatic spheres. The results show that insulin-positive clonal spheres can be isolated as early as the pancreatic endoderm stage as well as the pancreatic progenitor stage during the hESC to beta-cell lineage differentiation model and that they can be isolated only as early as the pancreatic progenitor stage during mouse embryogenesis. Further, pancreatic clonal sphere-forming cells isolated from the pancreatic progenitor stage in embryonic mice display multipotentiality, and those isolated at a later gestational age demonstrate self-renewal ability. These findings suggest that pancreatic precursors isolated from mouse embryonic time points have stem cell properties and that the pancreatic progenitor stage in hESC development may be the optimal time to capture and expand these stem cells and make large numbers of beta cells.

## Introduction

There are three types of mature cells in the adult pancreas: endocrine, exocrine and ductal, where endocrine cells constitute the beta cells (insulin-producing cells), alpha cells (glucagon-producing cells), delta cells (somatostatin-producing cells), gamma cells (pancreatic polypeptide cells), and epsilon cells (ghrelin producing cells). In addition to these mature cell types, it has been previously shown that there are multi-potential, proliferative pancreatic precursors in the adult mouse (Seaberg et al., 2004; Smukler et al., 2011; Arntfield and van der Kooy, 2013; Razavi et al., 2015; Brokhman et al., 2019) and human (Smukler et al., 2011). Seaberg et al., 2004 clonally derived these pancreatic precursors from mice using a previously established clonal sphere assay (developed to study brain stem cells *in vitro* (Reynolds and Weiss, 1992) and found that this rare population of pancreatic precursor cells exist at a frequency of 1 in 10 000 pancreatic cells. These pancreatic precursor cells express pancreatic genes, and although they could not extensively self-renew, they displayed multipotentiality by differentiating into endocrine cells (including beta cells, delta cells, and alpha cells), exocrine cells (amylase cells), and neurons. The beta cells that came from these isolated precursors expressed insulin, c-peptide, and Glut2, markers of differentiated beta cells. However, the one lineage these mouse pancreatic precursors did not differentiate into was ductal cells. Subsequently, our lab repeated these results for human pancreas, but in contrast to mice, clonal human pancreatic precursors can robustly self-renew and could differentiate into mature insulin-responsive beta cells *in vivo* after PMP sphere transplantation into diabetic mice, effectively lowering their blood glucose levels (Smukler et al., 2011). It was later confirmed that these PMPs, despite their capacity to differentiate into a low percentage of neurons *in vitro*, come from the PDX1 lineage, which is a specification marker of the endoderm (and are distinct from the neural crest lineage) (Arntfield and van der Kooy, 2013). Taken together, these precursor cells were originally named “pancreatic multipotent progenitors (PMPs)” instead of “pancreatic stem cells” due to their incomplete differentiation potential and the inability of the mouse-derived precursors to self-renew.

Our lab had previously also shown that not only do mature adult beta cells express insulin but also the immature PMPs express insulin as well (although PMPs do not express the mature beta cell marker Glut2, which is necessary for glucose responsiveness) (Smukler et al., 2011). This corroborates a more recent study showing through flow cytometry and RNA sequencing that there could be at least four subpopulations of insulin-producing cells, where all subpopulations have similar amounts of insulin granules but have varying degrees of capacity to respond to glucose and release their insulin, and that two out of four of these populations have an upregulation of genes associated with neurogenesis (Dorrell et al., 2016). Indeed, we have suggested that the differentiation of neurons from clonal PMPs points to the evolution of beta cells through the co-option of a neuronal transcription program (Arntfield and van der Kooy, 2011). Another study using single-cell mass cytometry also revealed different subpopulations of insulin cells, where the proliferation marker, Ki67, was significantly more prevenient in two of their clusters (Wang et al., 2016). Further still, some studies conclude that there are differences between the level of insulin protein and granularity between different insulin + subpopulations, where greater insulin and granularity levels reflect a greater ability for the cells to release insulin after glucose stimulation, and lower granularity reflects resistance to cell death (Benninger and Kravets, 2022). The combined data suggest that these subpopulations differ in maturity despite all still producing insulin at some level.

It is viewed traditionally that stem cells can self-renew during the organism’s lifetime and sit at the top of the lineage hierarchy (i.e., the stem cell proliferates to give rise to multiple differentiated cells in the organ *in vivo* and *in vitro*). However, there are cases where an organ or entire organism is not initiated by a true stem cell but by a transient progenitor cell with large and rapid proliferative capacity that forms the bulk of the tissue; and then sometime later during its lineage, a subset progenitor population acquires stem cell characteristics (van der Kooy and Weiss, 2000). “Set aside cells” of invertebrate insects, such as *drosophila* and of metamorphizing amphibians, are set aside during larvae development and are withheld from the differentiation processes of the late embryo, only to later make the tissues of the adult organism post-embryonically (Peterson et al., 1997).

Eye development initiates after the eye field evaginates from the neuroectoderm at embryonic day 8 (E8) in mice (Fuhrmann et al., 2014). However, it is not until the retinal pigment epithelium is completely specified at E11.5 that clonal retinal spheres that show consistent hallmarks of stemness (self-renewal and differentiation) emerge (Tropepe et al., 2000). For the brain, it was previously thought that the earliest time point at which neural stem cells (FGF-responsive definitive neural stem cells) emerged was at E8.5, which is a day after neural tissue formation begins (E7.5) (Tropepe et al., 1999). It was shown later that leukemia inhibitory factor (non-FGF dependent) primitive neural stem cells can indeed be isolated before neural tissue formation (as early as E5.5) and give rise to the later emerging definitive neural stem cells (Hitoshi et al., 2004), which then generate most of the adult brain. This study indicates that the point of emergence of an organ stem cell does not always correspond to the initial emergence of organ formation. This present study aims to elucidate when pancreatic stem cells emerge during embryonic development.

Lineage tracing experiments using *Pdx1-Cre*; ROSA-YFP and *Wnt1-Cre*; ROSA-YFP revealed that adult insulin + pancreatic multipotent progenitors (PMPs) originate from the endoderm PDX1 lineage (Arntfield and van der Kooy, 2013). However, the embryonic timepoint in which these precursor cells emerge has not been established, and unlike those isolated from adults, those that do emerge during embryonic development may have more capacity to self-renew.

At E8.5, PDX1 is first detected on the dorsal and ventrolateral side of the posterior foregut. As the pancreas begins to bud and embryological organogenesis progresses, PDX1 is ubiquitously expressed throughout the pancreas (Guz et al., 1995). During late gestation, PDX1 is progressively restricted to islet cells, and after birth, PDX1 is restricted to beta cells, a few delta cells, a few epithelial cells of the posterior stomach and the entire duodenal mucosa (Guz et al., 1995; Offield et al., 1996). PDX1 expression is necessary for NKX6.1 expression (Sander et al., 2000). Nkx6.1 is required for the beta-cell lineage to continue to the endocrine progenitor stage and is instigated at E10.5, marking the beginning of the pancreatic progenitor stage of embryonic mouse pancreatic development (Sander et al., 2000). Development of the ductal/endocrine lineages is associated with loss of *Ptf1a* and maintenance of *Nkx6*.*1* expression (Schaffer et al., 2010).

Neurogenin3 (Ngn3) has been found to specify a multipotent progenitor that gives rise to the four endocrine cell lineages of the pancreas (Gradwhol et al., 2000). Ngn3 patterns multipotent progenitors by activating the endocrine genes (causing multipotent progenitors to gain endocrine and ductal competence), as well as by stimulating Notch signalling, which ultimately works to inhibit the Ngn3 protein and endocrine differentiation (Qu et al., 2013).

An upregulation of MAFA (mature beta-cell marker) through tyrosine kinase receptor AXL inhibition, ALK5 inhibition, and thyroid hormone has been shown to drive beta-cell maturation during hESC differentiation (Rezania et al., 2014). TGF-β type I receptor inhibitors and vesicular monoamine transporter 2 inhibition also have been shown to induce NEUROG3 expression (Sakano et al., 2014).

We followed Korytnikov and Nostro’s (2016) protocol to differentiate hESCs through to the equivalent of the stages of mouse pancreatic development, which are definitive endoderm (DE), posterior foregut (PF), pancreatic endoderm (PE), and pancreatic progenitor (PP). The cells of the DE are CXCR4 expressing, while PDX1 expression first emerges at the PE stage. The co-expression of PDX1 and NKX6.1 marks the PP stage, and this stage can still give rise to all three of the endocrine, exocrine, and ductal stages. The flow of these stages follows early mouse pancreatic development by using specific amounts of various factors (Korytnikov and Nostro, 2016). According to this protocol, the next stage toward a monohormonal beta-cell population is called the endocrine progenitor (Ngn3 positive cells) stage, in which cells are finally restricted to becoming endocrine cells. This is then followed by a few more stages (unpublished) until their lab creates a population of mature beta cells.

Using the beginning of this specific protocol to differentiate the ins-GFP hESC line towards the definitive endoderm (DE), posterior foregut (PF), pancreatic endoderm (PE), and then the pancreatic progenitor (PP) stages (Korytnikov and Nostro, 2016), we have found that clonal human sphere-forming cells that generate clonal insulin + spheres can be isolated at the hESC-PE stage and hESC-PP stages, which correspond to mouse embryonic day 8.5 (E8.5) and E10.5, respectively. Insulin + cells within hESC-derived PE-generated spheres are initially polyhormonal for insulin and glucagon but lose their glucagon expression following 14 days of re-exposure to PE stage media, which is the earliest that monohormonal insulin + cells have been seen during hESC to beta cell differentiation.

In addition, using PDX1 expression as a pancreatic specification marker, we isolated pancreatic sphere-forming cells from *Pdx1-Cre*; ROSA-YFP lineage tracing transgenic mice at the embryonic PP (E11) stage but not from the earlier E8.5 PE stage. Some progenitors within PDX1-YFP+ spheres differentiated into insulin + beta cells as well as other endocrine cell types in 1% serum, suggesting that embryonic-derived pancreatic spheres are multipotential like their adult counterparts. At later stages (E16), PDX1-YFP+ spheres passage at least three times *in vitro*, reliably demonstrating the bonafide stem cell quality of self-renewal. Based on these results, we suggest that pancreatic precursors emerge early in embryonic development and then acquire stem cell characteristics of self-renewal at some point during their lineage.

## Materials and methods

### Animals

Mice used in this study included ROSA-YFP/R26R-EYFP (Jackson labs) (Srinivas et al., 2001), Hemizygous (*Pdx1*-Cre)6Tuv/J (abbreviated as *Pdx1*-Cre; Jackson labs) (Hingorani et al., 2003), and *Pdx1*-Cre*;* RosaYFP.

### Immunocytochemistry

Single mouse and hESC-derived pancreatic colonies were fixed in 4% paraformaldehyde (PFA, Sigma) for 10–11 min at room temperature after being plated down. They then were incubated for 1 h in blocking solution containing 1X PBS, 5% normal goat serum (NGS, Jackson Immunoresearch) and 0.3% Triton™ X-100 (Sigma) to permeabilize the cells. Primary antibodies were incubated overnight at 4°C in 5% NGS in 1X PBS, and secondary antibodies were incubated for 1 h at room temperature in 5% NGS in 1X PBS. Nuclei were stained with Hoechst (Sigma, 33,258, 10 μg/mL or 1:1,000) for 10 min at room temperature. Images were taken using an inverted fluorescence AxioVision Zeiss UV microscope with an AxioCam MRm camera and AxioVision v4.6 imaging software (Zeiss) or Olympus Fluoview FV1000 confocal laser scanning microscope. Primary antibodies include anti-insulin, mouse monoclonal (1:1,000, #I2018, Sigma); anti-insulin, guinea pig polyclonal (1:200, #7842, Abcam), anti-glucagon, mouse monoclonal IgG_1_ (1:2,000, #G2654, Sigma), anti-GFP, chicken polyclonal IgY (1:1,000, #GFP-1020, Aves), anti-C-Peptide, rat monoclonal (concentrated version, 1:1,000, #GN-1D4, Developmental Studies Hybridoma Bank).

### hESC cell culture

*hESC* to beta cell cytokine differentiation was performed until the pancreatic progenitor stage, according to Korytnikov and Nostro (2016). All H1 hESC-derived DE, PF, PE, and PP cells were provided directly by the Nostro lab. HES3/ins-GFP reporter hESC-derived DE, PF, PE and PP cells (Micallef et al., 2012) were either provided directly by the Nostro lab or isolated independently according to their protocol (Korytnikov and Nostro, 2016). The ins-GFP hESC cell line was approved for use by the Murdoch Children’s Research Institute (ABN 21 006 566 972) of the Royal Children’s Hospital in Australia and The Governing Council of the University of Toronto.

When we independently differentiated the HES3/ins-GFP cells, the original vial was provided by Dr. Christina Nostro, expanded upon and frozen down into multiple vials for later use. To start the differentiation, a frozen vial was thawed across about 3 wells of a 6-well plate using hES media. The hES media components included 200 mL of DMEM/F12, 50 mL of knockout serum replacement multispecies, 3.2 mL of sodium bicarbonate 7.5% solution, 2.5 mL of Glutamine, 2.5 mL of MEM non-essential amino acids solution, 2.5 mL of penicillin/streptomycin, 0.5 mL sodium pyruvate solution, 0.250 mL of 2-mercaptoethanol, and 7 ng/mL FGF2 (which was added fresh right before feeding cells each time). These materials are listed in Korytnikov and Nostro (2016). They were expanded upon until at least two full 6-well plates were covered with a monolayer of pluripotent hESCs. Their pluripotency was checked with immunostaining for pluripotency markers such as October 4 (data not shown). Incubation with TryplE for 3 min was always used when cells to lift cells from the plate for passaging/expansion and freezing. For freezing, freezing media containing 40% ES qualified (Wisent) FBS, 50% hES media, and 10% DMSO were used.

On Day 0 (when hESCs are ready to start the differentiation process), 1% L-Glutamine, 3 μL/mL of diluted monothioglyrcerol (MTG), 100 ng/mL Activin A, and 2 μM CHIR99021, all diluted in RPMI were used to feed cells. On Day 1, 1% L-Glutamine, 3 μL/mL of diluted MTG, 100 ng/mL Activin A, 5 ng/mL FGF2, and 10uL/mL ascorbic acid, all diluted in RPMI were used to feed cells. By Day 3, cells have reached the DE stage. On Day 3, 1% B27, 3uL/mL of diluted MTG, 1% L-Glutamine, 50 ng/mL FGF10, and 0.75 μM Dorsomorphin, all diluted in RPMI media were used to feed cells to bring cells to D6 (PF stage). On D6, 1% L-Glutamine, 10 μL/mL ascorbic acid, 1% B27, 50 ng/mL Noggin, 50 ng/mL FGF10, 0.25μM SANT1, and 2 μM retinoic acid (RA), all diluted in DMEM HG media were used to refeed cells until D8 at which point cells transition to the PE stage. On D8, 1% L-Glutamine, 10 μL/mL ascorbic acid, 1% B27, 50 ng/mL Noggin, 100 ng/mL hEGF, and 10 mM Nicotinamide, all diluted in DMEM HG were used to refeed cells until Day 13, at which point cells transition the PP stage.

Ascorbic acid was reconstituted in water at 10 mM stock concentration and stored at −20°C. Nicotinamide was reconstituted in IMDM media and aliquoted into ready-to-use vials at a stock concentration of 1,637.7 mM and stored at −20°C. RA was stored in aliquots at −80°C. Feeding of cells that involved RA was done in the dark. Noggin, L-Glutamine, B27, FGF2, and hEGF were stored at −20°C. All other components were stored at 0°C.

At each stage of differentiation, DE, PF, PE, and PP cells underwent a clonal sphere-forming assay, which is a highly reproducible assay (Reynolds and Weiss, 1992; Coles-Takabe et al., 2008; Pastrana et al., 2011). Stem cell frequency is calculated based on the number of clonal spheres generated from a given tissue sample. Briefly, cells were lifted and dissociated with TryplE Express (Gibco). Incubation times with TryplE Express were as follows: DE: 3 min, PF: 3 min, PE: 7 ½ mins, PP: 3 min), and centrifuged at 350 g for 5 min in 1X PBS and 10 μM Rock Inhibitor (RI). Cells were resuspended in defined serum-free medium (SFM) (Tropepe et al., 1999) containing 10 ng/mL basic fibroblast growth factor (FGF2) (Sigma), 20 ng/mL EGF (Sigma), 2 mg/mL heparin (Sigma), 1 × B27 Supplement (Gibco-BRL) and 10 μM Rock Inhibitor (RI). Cells were counted using Trypan Blue exclusion and hemocytometer and immediately replated at a low clonal density in SFM/FGF2/EGF/heparin/1xB27/RI either at clonal density (Coles-Takabe et al., 2008) in 24-well ultra-low attachment plates (3473/07-200-600, Fisher/Sigma). Specifically, DE- and PP-derived single cells were plated at 10 cells/μL because sphere production from these stages was minimal and plating at 10 cells/μL density maintained clonality where no fusion of spheres occurred but sphere count was not too sparse as to be difficult to count and obtain a sufficient number of spheres. PF- and PP-derived single cells were plated at 1 cell/μL because too many spheres are produced when they are plated at 10 cells/μL, which results in sphere fusion and a loss of clonality. Reducing the plating density to 1 cell/μL was needed to ensure clonality for PF- and PE-derived spheres in the clonal sphere-forming assay.

Cells then were incubated for 1 week without moving to reduce the incidence of non-clonal colonies (Coles-Takabe et al., 2008). To assess differentiation potential, single colonies were removed from the aforementioned mitogen-containing media and transferred to wells coated with either Geltrex or Laminin 411, in SFM containing Stage 3 media and grown for an additional 1 or 14 days before fixation and analysis. To ensure an accurate assay of the sphere progeny, each well of a plate (Nunc) contained only one clonal DE-, PF-, PE-, or PP-derived colony.

### Mouse embryo cell culture

The morning of the first appearance of vaginal plugs was regarded as 0.5 days post conception (dpc). *Pdx1*-Cre*;* RosaYFP E11 was dissected under a dissection microscope (light microscope), where the location of pancreatic bud was located using the Greggio et al. (2014) video, where they showed a dissection of E10.5 pancreatic bud. In brief, the embryonic dissection was performed in PBS, and the digestive tract was identified and removed. From the successful isolation of an intact digestive tract, the pancreatic bud was identifiable and was isolated using forceps.

Next, the dorsal pancreatic bud that was isolated from E11 tissue was triturated in TryplE 10-20X. The resulting single cells from E11were centrifuged in 1X PBS or SFM/FGF2/EGF/heparin/1xB27 (both had produced similar results) and resuspended in SFM/FGF2/EGF/heparin/1xB27. Following trituration and centrifugation, cells were counted with Trypan Exclusion and plated at a cell density of 10 cells/µL to ensure clonality in SFM/FGF2/EGF/heparin/1xB27 in ultra-low adhesion plates. The plates then were incubated for 1 week without moving. This part constitutes the sphere-forming assay, which is a highly reproducible assay (Reynolds and Weiss, 1992; Coles-Takabe et al., 2008; Pastrana et al., 2011). Stem cell frequency is calculated based on the number of spheres generated from a given tissue sample.

### Statistics

Data are presented as means ± standard errors of the means (SEM). Statistical analyses were performed using GraphPad Prism 9 (GraphPad Software Inc.). Student’s t-tests (two-tailed) were performed for statistical analysis between two groups. A one-way ANOVA or two-way ANOVA with a Holm-Sidak *post hoc* test (pairwise or *versus* control comparison) was used when three or more groups were compared. Sample sizes (N) and p-values are provided in the figure legends. Statistical significance was set at p < 0.05.

## Results

### Pancreatic multipotent progenitors emerge at the human pancreatic endoderm stage

To determine the embryonic timepoint in which pancreatic stem cells emerge using the hESC to beta cell model, hESCs were differentiated towards the DE, PF, PE, and PP stages as previously described (Korytnikov and Nostro, 2016). All H1-derived DE, PF, PE, and PP cells were obtained fresh directly from the Nostro lab, where they frequently ensure the identity of the cells through flow cytometry at these four initial stages. Although we obtained some HES3/ins-GFP-derived DE, PF, PE, and PP cells directly from the Nostro lab as well, this was only at the beginning of our project for a proof of concept. We were able to later independently differentiate the HES3/ins-GFP line to continue with the majority of our experiments, and we were able to perform flow cytometry to qualify our own differentiation. Through our own differentiation HES3/ins-GFP cells transitioned to the DE stage with about 93% of cells co-expressing specific DE markers CXCR4 and CD117; the PE stage, where about 96% of cells were PDX1 + NKX6.1-; and to the PF stage where about 95% of cells were PDX1 + NKX6.1+ (Supplementary Figure S1). This shows we were able to obtain relatively pure populations of the desired stages.

The cells, once they reached each of the first four stages, were dispersed and passed through a 40 μL cell strainer before plating at a low cell density in low cell-adhesion 24-well plates. Specifically, DE- and PP-derived cells were plated at 10 cells/μL because sphere production from these stages was minimal and plating at 10 cells/μL density maintained clonality. However, PF- and PE-derived cells were plated at a much lower density of 1 cell/μL because too many spheres are produced when they are plated at 10 cells/μL or even at 5 cells/μL, which results in sphere fusion and a loss of clonality. Reducing the plating density to 1 cell/μL ensured clonality when cells from the PF and PP stages were plated in the clonal sphere-forming assay. Coles-Takabe et al. (2008) were able to show that by plating at low density (greater than single cells per well), spheres can grow clonally. After sphere growth, stem cell frequency from the original hESC monolayer culture at each of the four stages of differentiation was determined based on the number of spheres generated within the sphere assay.

**FIGURE 1:**
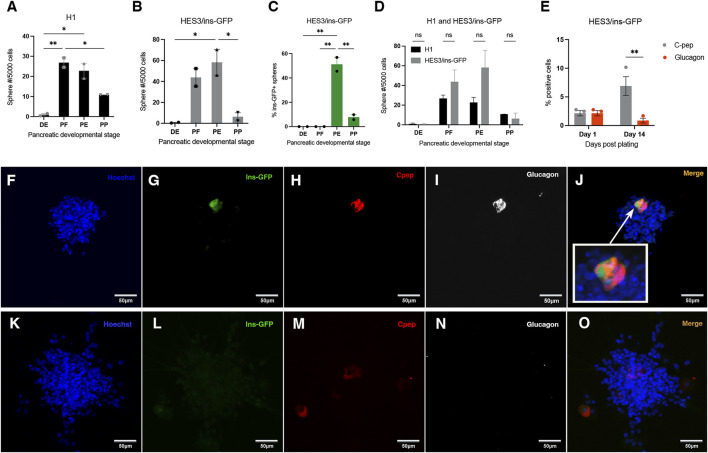
Spheres are generated from H1 and ins-GFP hESCs as they progress through the pancreatic lineage, and ins-GFP + spheres first emerge at the pancreatic endoderm stage of development. **(A, B)** Quantification of sphere number after a clonal sphere formation assay of H1 or ins-GFP hESC-derived definitive endoderm (DE), posterior foregut (PE), pancreatic endoderm (PE) and pancreatic progenitor (PP) cells reveals that both H1 and ins-GFP hESC-derived DE, PF, PE, and PP cells give rise to spheres. The different frequencies of spheres generated across each stage follow a similar pattern between H1 and ins-GFP cell lines, where the PF and PE stages generate the highest frequencies and are several-fold higher than the DE and PP stages. **(A)** H1: one-way ANOVA F_(3,4)_ = 28.85, P = 0.0036; Holm-Sidak’s posthoc test, * = p < 0.05; N = 2, where N represents the number of biological replicates, and there were 24 technical replicates for each differentiation stage, within each biological replicate. **(B)** ins-GFP: one-way ANOVA F_(3,4)_ = 13.46, p = 0.0148; Holm-Sidak’s posthoc test, * = p < 0.05; N = 2 biological replicates). Black bars (for H1 hESC cell line) or grey bars (for ins-GFP hESC cell line) indicate the average total sphere frequency per 5,000 cells ( ± SEM). **(C)** The percentage of ins-GFP + spheres (out of the total average number of spheres generated from each stage) in the ins-GFP cell line suggests that no insulin positivity is seen prior to the PE stage. Almost 50% of all spheres generated at the PE stage are ins-GFP+, which reduces to about 8% at the PP stage. One-way ANOVA F_(3,4)_ = 66.64, p = 0.0007; Holm-Sidak’s posthoc test, * = p < 0.05; N = 2, where N represents the number of biological replicates, and there were 24 technical replicates for each differentiation within each biological replicate. Green bars indicate the average number of spheres out of the total number of spheres that are ins-GFP+ ( ± SEM). **(D)** Comparison between H1 and HES3/ins-GFP cell line generation of DE, PF, PE, and PP spheres reveals that there is no significant difference between the frequency of spheres generated between H1 and HES3/ins-GFP cell lines. Two-way F_(2,6)_ = 0.1321, p = 0.8787; Holm-Sidak’s posthoc test, * = p < 0.05; N = 2, where N represents the number of biological replicates for each cell line, and there were 24 technical replicates for each biological replicate for each cell line. **(E)** Quantification based on immunohistochemistry of ins-GFP+, C-peptide+ (cpep+) and glucagon + cells within spheres after maintaining ins-GFP + spheres on Laminin 411 and in PE media for 1 or 14 days. Cpep + cells overlap with ins-GFP + cells at 1 day post-plating. Since only one biological replicate was performed, the results are not definitive. However, there is a trend where all cpep + cells overlap with glucagon 1 day after plating, but no or limited glucagon expression is seen in surviving cpep + cells by the 14th day. One-way ANOVA F_(1,4)_ = 19.17, P = 0.0119; Holm-Sidak’s posthoc test, * = p < 0.05; N = 1, where N represents the number of biological replicates, and there were 2 technical replicates (number of spheres per condition), within the biological replicate, similar sized spheres (80–100 µm) per condition. **(F–J)** Representative images of immunohistochemistry staining of ins-GFP + PE-derived spheres after maintaining them in PE media for 1 day. Arrows indicate that labelled cells co-express cpep and glucagon, indicating they are polyhormonal and are, therefore, immature. They also all express ins-GFP, where some expressed it strongly (cells toward the left of the group of cells), and some expressed it weakly (cells on the right of the group of cells). The weak expression in some of the cells might be due to the instability and half-life of ins-GFP. **(K–O)** Representative images of immunohistochemistry staining of ins-GFP + PE-derived spheres after maintaining them in PE media for 14 days. While some cpep + cells remain, glucagon never overlaps with them, indicating that these cells are no longer polyhormonal. The absence of glucagon + cells, in general, indicates that PE media promotes the beta cell lineage rather than the alpha cell lineage.

We used DE, PF, PE, and PP cells from the H1 and HES3/ins-GFP lines (the latter will be referred to as ins-GFP from now on), for the initial primary sphere assay to compare sphere number results between them. The H1 and ins-GFP cell lines require the same cytokines, with the same exposure times, during hESC differentiation (Nostro et al., 2015; Nostro et al., 2011). Therefore, drastically different outcomes in sphere generation between the 2 cell lines would not be expected. The frequency of hESC-derived PF and PE-generated spheres from the ins-GFP cell line was about 1.6 and 2.6-fold higher than that of H1, respectively (Figure 1D). However, the overall pattern of sphere formation between the different stages was similar between H1 and ins-GFP lines (Figures 1A, B, D). The varying frequencies of spheres generated across the stages indicate that each stage carries varying proportions of proliferating sphere-forming cells.

The ins-GFP reporter line was used to quickly visualize whether any spheres generated were ins-GFP+ and, therefore, part of the pancreatic lineage. About 50% of all spheres generated from the PE stage and about 10% of all spheres generated from the PP stage were ins-GFP+ (Figure 1C). Since insulin is a marker of adult pancreatic precursor cells (Smukler et al., 2011), insulin + cells within the sphere isolated at these early developmental time points are predicted to originate from an insulin + pancreatic stem cell or progenitor. There is more than a four-fold reduction in the percentage of ins-GFP + sphere formation between the PE and PP stages, as well as a significant reduction in the frequency of the total number of spheres (Figures 1A–C). We suggest that the difference in environmental factors between the two stages affects the survival and/or proliferation of both ins-GFP + spheres and ins-GFP- sphere-forming cells. To start characterizing whether these ins-GFP + cells in these primary spheres had stem properties, we assessed their ability to self-renew by bulk passaging the ins-GFP+ and ins-GFP- PE primary spheres. Upon secondary sphere formation, we saw that 77% of secondary spheres were ins-GFP+, suggesting that most of the insulin + cells (within the primary sphere) are precursors that are sphere-initiating cells and, therefore, have stem-like qualities (Supplementary Figures S2A, B). The other 20% of secondary spheres that do not contain ins-GFP + cells were either derived from the ins-GFP- primary spheres, which in turn were derived from a non-pancreatic lineage, or they were from ins-GFP + primary spheres and the ins-GFP + stem or precursor cell lost its insulin expression during secondary sphere growth.

### hESC-derived insulin + precursors are initially polyhormonal but then differentiate into monohormonal insulin + cells

Similar-sized single ins-GFP + PE spheres were incubated with PE stage media on either Geltrex or laminin for 14 days. We observed that the survival of cells within clonal spheres was significantly greater when plated on laminin compared to when plated on Geltrex (Supplementary Figure S3A). The cells also remained closer together on laminin than on Geltrex, but the results were not significant (Supplementary Figure S3B). Future experiments assessing the multipotential nature of PE-generated ins-GFP + spheres should focus on using laminin as the extracellular matrix.

Polyhormonal cells emerge starting at E8.5 in the developing mouse pancreas and during the PE stage of hESC differentiation (Korytnikov and Nostro, 2016; Nostro et al., 2011; Nostro et al., 2015; Gittes et al., 1996). We wanted to determine if the ins-GFP + cells within spheres generated from the PE stage are polyhormonal for glucagon. Due to the enhanced survival (Supplementary Figure S3A) and compactness (Supplementary Figure S3B; although the difference in compactness was not significant) of the cells in our initial observation with laminin *versus* Geltrex and due to other studies showing that the prevention of cell migration/spreading by laminin (*versus* Geltrex) in the plate promote pancreatic identity (Mamidi et al., 2018), the PE generated spheres plated on laminin were further analyzed. Therefore, in this case, ins-GFP + spheres were stuck down on the laminin extracellular matrix in the presence of PE stage media for 1 or 14 days before fixation and immunostained for C-peptide, glucagon and GFP. After 1 day, 100% of the total number of ins-GFP+/C-peptide + cells in the spheres analyzed were co-labeled with glucagon (Figure 1E). This indicates that all insulin + cells within PE-generated spheres are polyhormonal with glucagon, and that therefore, they are immature and may be primed to differentiate into the alpha cell lineage, according to Nostro et al. (2015). All C-peptide + cells also expressed ins-GFP, however, some cells expressed GFP weakly and other cells expressed GFP strongly (Figures 1F–J).

Following a 14-day incubation in Stage 3 media, the C-peptide + cells in ins-GFP + spheres either no longer expressed glucagon or expressed significantly less glucagon (Figures 1E–O). Although no lineage tracing was done in this experiment, all C-peptide + cells in hESC-derived PE spheres were initially polyhormonal for glucagon. No single labelled insulin + or single labelled glucagon + cells were seen when spheres were fixed and stained 1-day post-fixation (Figure 1E). When hESC PE generated ins-GFP + spheres were plated on laminin and re-exposed to stage 3 media for a prolonged period of 14 days, C-peptide + cells were still seen, and many of these C-peptide + cells no longer had glucagon expression overlapping them, and those that had still glucagon expression overlapping them had faint levels of this glucagon expression (compared to what had been previously co-expressed in the initial sphere 1-day post-fixation) (Figure 1E).

Although the above results are not conclusive due to the absence of a lineage tracing experiment to trace the descendants of the polyhormonal cells, they suggest, indirectly, that these cells, given enough time in PE Stage media, may show a trend of changing their identity towards monohormonal insulin + progenitors. However, after 14 days, C-peptide also was less strong in expression as well, indicating that optimizing the maintenance of beta cell identity may need further studies.

### Pancreatic stem cells can be isolated at the mouse PP stage, as well as later from E16 mice

**FIGURE 2:**
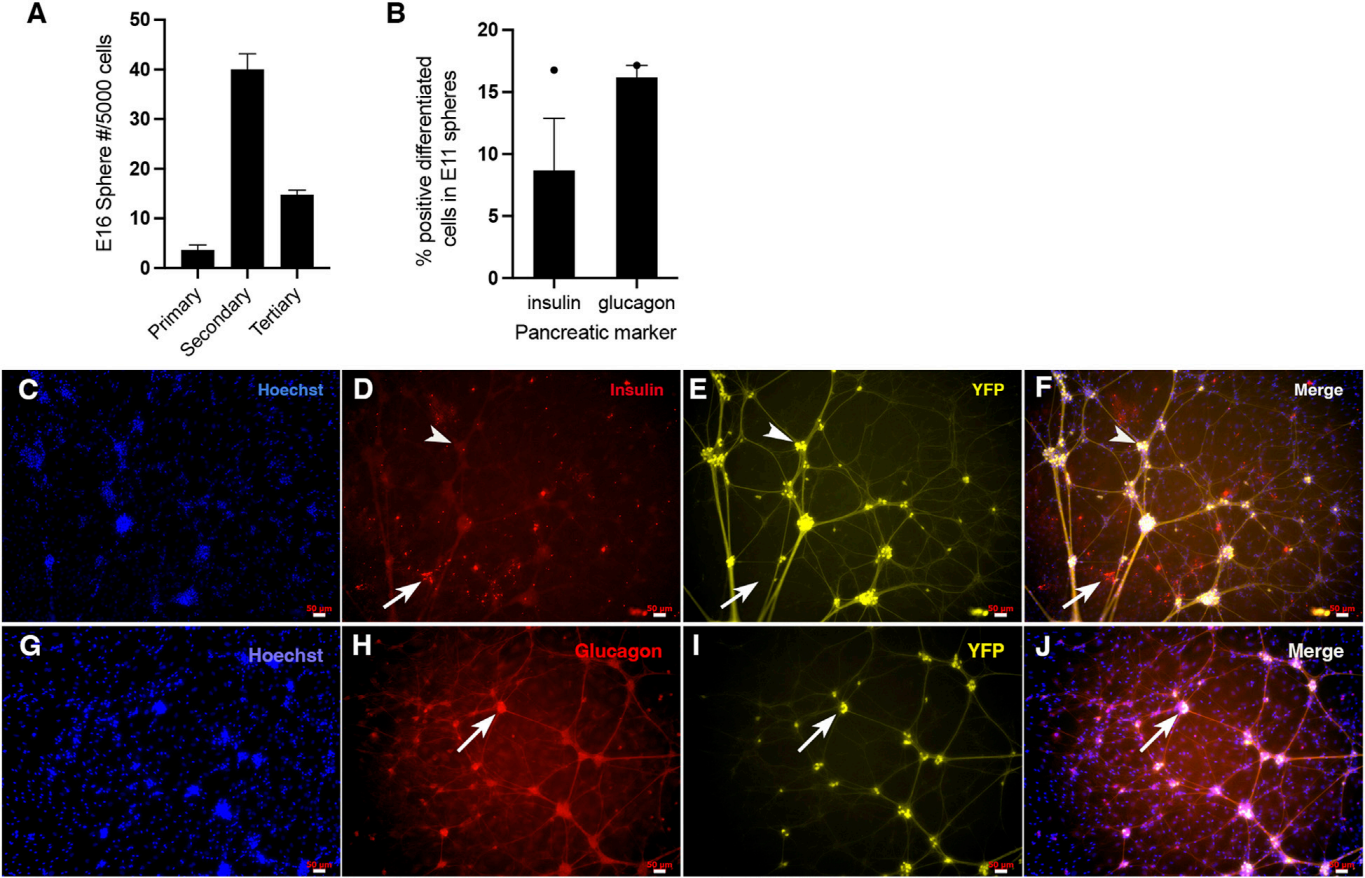
Single cells from the embryonic pancreas give rise to clonal pancreatic spheres that have the potential to differentiate into multiple pancreatic cell types, as well as self-renew. **(A)** E16 *Pdx1*-Cre*;* RosaYFP mouse spheres self-renew. Passaging of all 43 primary PDX1-YFP+ spheres gave rise to 482 secondary PDX1-YFP+ spheres (11-fold increase), and passaging of these 482 spheres gave rise to 356 tertiary spheres (revealing a 4-fold increase from primary PDX1-YFP+ spheres to tertiary spheres and 2.6-fold decrease from secondary spheres to tertiary spheres. One biological replicate was done for this experiment. The preliminary evidence suggests that E16 pancreatic sphere-initiating cells have a trend of being able to undergo symmetric self-renewal within primary spheres and asymmetric self-renewal within secondary spheres. **(B–J)** Isolation of the *Pdx1*-Cre*;* RosaYFP pancreatic anlage at E11 yielded homogenous PDX1-YFP+ spheres, confirming they are of pancreatic origin. After differentiating single PDX1-YFP+ spheres on Matrigel in the presence of 1% serum, differentiated progenitors within pancreatic spheres were immunostained for insulin and glucagon (separately), imaged using a fluorescent microscope and quantified. The presence of separate glucagon and insulin-containing cells confirms the spheres are of pancreatic identity. **(B)** About 8.5% of the progenitors within the spheres differentiated into insulin + cells (N = 1, where N represents the number of biological replicates and there were 2 technical replicates (number of spheres from the biological replicate) and about 16% of the progenitors within the spheres differentiated into glucagon + cells (N = 1, where N represents the number of biological replicates, and there were 3 technical replicates (number of spheres per condition) from the biological replicate). The preliminary evidence suggests that the E11-derived spheres tend to be multipotential for insulin+ and glucagon + monohormonal cells. **(C–F)** Representative images of Hoechst, insulin, and YFP staining of differentiated sphere progenitors, where YFP indicates that spheres are from PDX1 origin. Insulin + cells were present after E11 PDX1-YFP+ sphere differentiation. The arrow indicates an example of a few insulin-labelled cells. All insulin + cells were not YFP labelled, indicating that the PDX1 lineage marker turned off in these cells or that YFP expression had become too faint to see once these insulin + cells underwent differentiation. A separate arrowhead indicates insulin-/YFP + cell clusters with processes. These cells could be neurons, suggesting that these particular neurons are from the PDX1 lineage and that the lineage tracing marker continued to be expressed in these cells as the cells were differentiating. **(G–J)** Representative images of Hoechst, glucagon and YFP staining of differentiated sphere progenitors, where YFP indicates that spheres are from PDX1 origin. The arrow shows an example of a cluster of glucagon-labelled cells. Within this cluster, it appears that some glucagon + cells are co-labelled with YFP strongly, while some may not be (cells toward the left of the cluster). This indicates, as previously mentioned for insulin, that the PDX1 lineage marker turned off in these cells or that YFP expression had become too faint to see once these glucagon + cells underwent differentiation. It also appears that during differentiation, faint, thin YFP + processes have extended from these glucagon cells. However, because they are significantly fainter and less robust than the processes coming from the non-insulin cell clusters in C-F (presumably neurons), what appears to be the faintly labelled YFP processes coming from glucagon + cells in G-J may indicate an artifact. Alternatively, the glucagon + cells could be overlapping with (unlabelled) neurons.

To determine whether clonal sphere-producing pancreatic precursors first emerged at the PE stage in mice as they did in the hESC to beta cell model, we dissected the developing pancreas at E8.5-9, and single cells were cultured in a sphere-forming assay. After sphere growth, stem cell frequency in the original tissue sample was determined based on the number of spheres generated within the sphere assay. To ensure that the spheres obtained are from pancreatic PDX1 lineage origin, we utilized the early pancreas/duodenum specification marker PDX1 and the *Pdx1*-Cre*;* RosaYFP lineage tracing mouse. Homogenously labelled PDX1-YFP+ spheres from mouse E8.5-10 could not be isolated. However, homogenously labelled PDX1-YFP+ spheres were isolated at E11, which corresponds to the pancreatic progenitor developmental stage. Single similarly sized PDX1-YFP+ spheres isolated from the E11 PP stage were plated into a sphere differentiation assay with 1% serum and were immunostained for GFP and either insulin or glucagon. About 16% of progenitors within the sphere differentiated into glucagon + cells, and a separate 8.5% of the progenitors differentiated into insulin + cells (Figure 2B). At the PP stage, progenitors are known to be able to differentiate into three lineages: endocrine, exocrine, and duct cells (Gu et al., 2002). However, due to the limited number of spheres obtained, we did not test for the presence of exocrine and duct cells (via immunocytochemistry), and therefore, it remains unknown whether the precursors isolated at this stage are truly multipotential. Since previously it was shown that the adult mouse PMP could differentiate into various endocrine and amylase cells (Seaberg et al., 2004; Smukler et al., 2011; Arntfield and van der Kooy, 2013), it might be the case for E11 precursors as well, but this is not conclusive. Nonetheless, E11 cells having the ability to differentiate into monohormonal glucagon and insulin + cells indicates that the isolated E11 spheres are from pancreatic origin and that there is a trend toward possible multipotentiality of its progenitors. In the differentiation assay following sphere growth for E11 spheres, there may still be proliferation going on during the differentiation for E11 (Figure 2B), and if the cells are fully differentiating, we would want to stain for the proliferation marker ki67 to make sure proliferation is not continuing. However, this study was unable to conduct this experiment due to limited spheres.

Figures 2C–J shows clearly labelled differentiated glucagon + cells and insulin + cells derived from E11 mouse pancreatic anlage, revealing that pancreatic stem cells do derive from E11 mouse pancreas. Staining for glucagon and insulin was done separately for different spheres due to the availability of reliable antibodies. Therefore, we could not determine whether or not there was any insulin+/glucagon + co-expression in differentiated spheres. Since the mice used in this experiment were *Pdx1*-Cre; Rosa YFP (where YFP indicates that the cell was derived from the PDX1 pancreatic lineage regardless of whether or not that cell is currently producing PDX1), all cells that were fully differentiated or were in the process of differentiation should also express YFP. The insulin + cells from the E11 differentiation assay were not YFP+ (Figures 2C–F). This may indicate that the PDX1 lineage marker turned off in these cells during differentiation or sphere growth or that YFP expression had become too faint to see once these insulin + cells underwent differentiation. Some of the glucagon + cells from the sphere differentiation assay were not labelled with YFP and some were labelled with YFP strongly (Figures 2G–J). Again, for the glucagon+/YFP- cells, either the lineage marker had turned off during glucagon differentiation, or YFP became too faint to see once differentiation occurred. It has been previously shown that the *Pdx1*-Cre; Rosa YFP mouse strain can undergo transgene silencing (Arntfield and van der Kooy, 2013), which could occur at any stage *in vivo* or *in vitro*. It is undetermined if the differentiated insulin and glucagon cells were actively expressing PDX1 in these cells.

It is interesting to note that there are also YFP + cells with processes (Figures 2C–F). Although this indicates that the PDX1 lineage gives rise to neurons, the differentiated insulin + cells are distinct cells without processes. This aligns with previous studies from our lab (Smukler et al., 2011; Arntfield and van der Kooy, 2013; Brokhman et al., 2019), which reported that adult mouse PMPs (from the PDX1 lineage) also give rise to both beta cells and a small number of separate neurons that are also distinct and separate from the neural crest lineage.

Some glucagon + cells appear to overlap with cells that may have processes (Figures 2G–J). It could be that these glucagon + cells (that do not have processes themselves) are overlapping but not double-labeled with the neurons. Another explanation could be that since these YFP + processes from glucagon + cells are significantly fainter and less robust than the processes coming from the non-insulin cells (presumably neurons) in C-F, the faintly labelled YFP processes coming from glucagon + cells in G-J may indicate an artifact. Due to the limited number of spheres, the passaging capability was not tested.

Homogenous PDX1-YFP+ spheres also were isolated at E16. Passaging of E16 primary pancreatic spheres resulted in an ∼11-fold increase in the number of secondary spheres compared to the number of primary spheres (Figure 2A). This suggests that the original sphere-forming cells of the primary spheres have self-renewal stem cell properties and, further, that this pancreatic stem cell (which was the origin of the clonal sphere) must have engaged in multiple rounds of symmetric division, expanding the stem cell population, and thereby expanding the number of secondary spheres we see *in vitro*. There were fewer tertiary spheres than secondary spheres after secondary sphere passaging (Figure 2A). This suggests that after the earlier pancreatic stem cell symmetric expansion, the stem cells underwent some proliferative exhaustion, whereby proliferation reduced, switching from symmetric division to asymmetric division during secondary sphere growth, reducing the number of tertiary spheres seen *in vitro* (Figure 2A). Although hESC-derived PE spheres observed limited self-renewal, most secondary spheres were insulin+ (Supplementary Figure S2), suggesting that the starting sphere-forming cell is insulin+ (or less likely, that insulin + cells turned on after clonal proliferation commenced).

## Discussion

**FIGURE 3:**
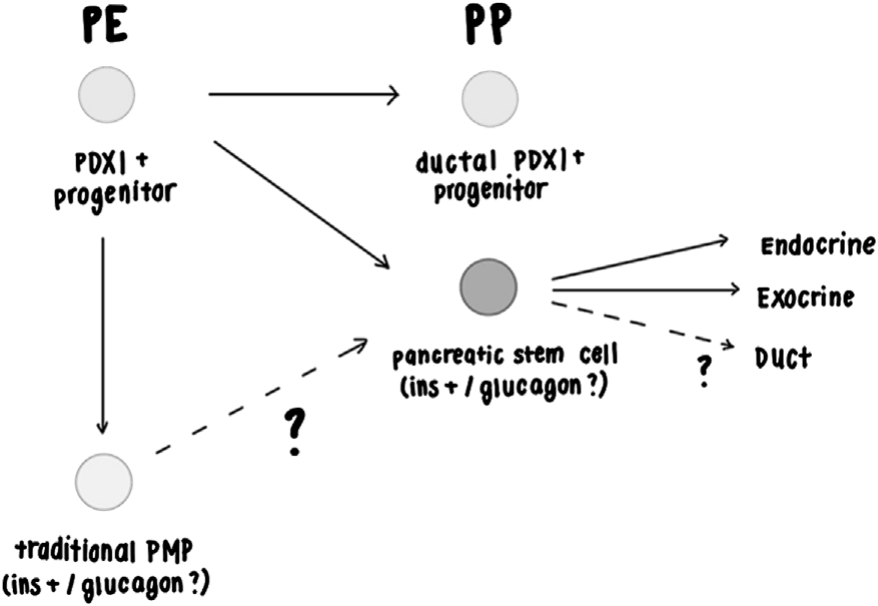
Proposed model of pancreatic stem cells during development. The model suggests that pancreatic precursor populations can be isolated at the hESC PE and PP stage and from the mouse PP stage based on the evidence presented in Figures 1, 2. Figure 2 showed that pancreatic precursors isolated at E16 self-renew, demonstrating that these precursors are stem cells and not a more restricted progenitor cell. Since adult mouse PMPs do not self-renew, we suggest that embryonically-derived pancreatic stem cells may be a different population of cells compared to adult PMPs or that embryonically-derived pancreatic stem cells lose the ability to self-renew in adults. Gu et al. (2002) had shown that PDX1 + progenitors give rise to ductal cells at mouse PP E10.5-12.5 stage, but rarely before or after this timepoint (Gittes and Rutter, 1992). Adult mouse (and human) PMPs don’t give rise to ductal cells (Smukler et al., 2011; Gu et al., 2002), suggesting that they may originate from the PE stage or after the PP stage. The PDX1+ mouse pancreatic spheres isolated at PP E10.5-12.5 in Figure 2 may have the capacity to differentiate into ductal cells, but this has yet to be determined. Figure 1 shows that within hESC-derived PE ins-GFP+ spheres, all GFP+ cells co-expressed C-Peptide and were also polyhormonal for glucagon expression. During hESC to beta cell differentiation PE stage PDX1+/NKX6.1- cells have the potential to give rise to polyhormonal cells when given a certain differentiation protocol, which in turn give rise to alpha cells during this (Korytnikov and Nostro, 2016; Nostro et al., 2011; Nostro et al., 2015). Past studies have shown that polyhormonal cells do not give rise to mature adult islets in mice (Gittes and Rutter, 1992; Offield et al., 1996; Herrera, 2000). Therefore, the isolated ins-GFP+/cpep+/glucagon+ polyhormonal cells isolated from hESC-derived PE stage spheres are predicted to be pancreatic stem cells that are set aside during development (and remain dormant until needed in adulthood) rather than stem cells that play an active role in creating the bulk of the pancreas or mature islet cells. It remains unclear whether traditional adult mouse insulin+ PMPs and embryonic mouse pancreatic stem cells that initiate the spheres are polyhormonal. Regardless of whether they are polyhormonal or monohormonal for insulin, we have shown that mouse-derived sphere progenitors that have been plated down in 1% serum differentiation conditions differentiate into separate monohormonal, mature cells (Figures 2B–F), (Seaberg et al., 2004; Smukler et al., 2011). Elucidating this, as well as whether the insulin+ cells in hESC-derived PP stage spheres and the insulin+ cells seen in the PE stage are independent populations, would allow us to determine the relationship between polyhormonal cells and pancreatic sphere-forming cells that have stem cell characteristics.

Pancreatic insulin+ clonal spheres first emerge at the hESC-derived PE stage of development and continue to emerge at a lesser frequency at the hESC-derived PP stage of development. Further, PDX1+ pancreatic spheres can be isolated at E11 in mice, which corresponds to the mouse PP stage in mouse, but not at E8.5, which corresponds to the mouse PE stage. The progenitors downstream of the mouse stem cell differentiated into separate insulin and glucagon cells, suggesting the possibility that they may be multipotential mouse embryonic PP-derived spheres (Figure 3). Mouse E16 pancreatic spheres were able to undergo multiple rounds of passaging, demonstrating that the sphere-initiating precursor cells have acquired self-renewing capability, which contrasts with the lack of self-renewal ability of the traditional precursors (PMPs) isolated from adult mice (Arntfield and van der Kooy, 2013; Seaberg et al., 2004; Smukler et al., 2011). We conclude that the isolation of pancreatic stem cells is possible at early mouse pancreatic developmental stages (E11) as well as later gestational periods (E16). Given that the pancreatic precursors isolated from E16 can passage, it warrants future investigation as to whether we could passage the E11-derived precursors as well and term these cells bonafide stem cells.

Mouse PDX1+ progenitors give rise to differentiated ductal cells between E10.5-12.5, before and after which point ductal cell emergence is extremely minimal (Gu et al., 2002). This time range corresponds to the PP stage. We would expect that pancreatic stem cells with the potential to differentiate into not only endocrine and exocrine lineages but also the ductal lineage would emerge during this time period. As mentioned, pancreatic precursors were isolated at the pancreatic progenitor (E11) stage from mice and have been shown to differentiate into beta cells and alpha cells. The presence of PDX1+ cells with the potential to give rise to ductal within the narrow time window of the pancreatic progenitor stage (Gu et al., 2002) suggests that if pancreatic stem cells that can give rise to duct cells exist, then they would most likely emerge at the pancreatic progenitor stage.

With regards to understanding whether a pancreatic stem cell exists and sits at the top of the hierarchy creating the entire pancreas, we predict the opposite, in that perhaps a subpopulation of PDX1+ transient progenitors emerge at the beginning of pancreas specification, the E8.5 PE stage to build the bulk of the pancreas, and are later induced (through extracellular signals from mesenchyme, or other nearby tissue derived from other germ layers) (Gittes et al., 1996), to acquire the stem cell characteristics of multipotentiality into all three pancreatic lineages, and self-renewal at the later PP stage (Figure 3).

It is undetermined whether some of the ins-GFP + cells from the earlier PE stage survive and constitute the population of PP emerging ins-GFP + cells that we observed in our data. We predict that this is not the case as other studies suggest that hESC-derived PDX1+/NKX6.1- PE cells give rise to polyhormonal cells, which then differentiate into mature alpha cells, while hESC-derived -NKX6.1+ PP cells can be induced to give rise to mature beta cells (Korytnikov and Nostro, 2016; Nostro et al., 2011; Nostro et al., 2015), which suggests PE-derived ins-GFP + cells and PP-derived ins-GFP + cells are different populations. In addition, polyhormonal cells in mice do not contribute to adult beta cells or glucagon cells (Gittes and Rutter, 1992; Offield et al., 1996; Herrera, 2000). If the embryonic polyhormonal cells we isolated are bonafide pancreatic stem cells) then we predict they are not playing a role in creating the bulk of the pancreatic tissue or mature islet cells but may be set aside during development until environmental stress occurs during adulthood.

Lineage tracing experiments to elucidate whether mouse E11 pancreatic progenitors give rise to the adult mouse pancreatic progenitors isolated in older studies and to more definitely determine whether polyhormonal cells from the hESC PE E8.5 give rise to monohormonal, functional insulin + cells would be worth future investigation. In addition to lineage tracing experiments, *in vivo*, transplantation of embryonic mouse- and hESC-derived precursor cells or their descended beta-cells into diabetic mice could be added to future studies investigating this topic further to make conclusions on their ability to reverse diabetes symptoms. This experiment would be analogous to the one Smukler et al. (2011) did, where adult mouse PMPs were transplanted into diabetic mice (streptozotocin-induced BalbC and NOD-Scid mice). Here, they showed that C-peptide levels, blood glucose levels, and weight loss recovery improved in PMPs transplanted mice comparable to those that received transplants of mouse islets. Having this additional experiment, transplanting embryonic pancreatic spheres and hESC-derived spheres, would help the research pave its way to the clinic.

Limitations of the sphere assay include the fact that it does not detect quiescent stem cells/progenitors, so our data cannot comment on the status of quiescent stem cells in the original tissue or culture. Another caveat is that the high FGF2 and EGF that are required can skew the fate of progenitors under differentiation conditions. Last, perhaps the most ideal method to ensure clonality *in vitro* is to plate a single cell/well, however, sphere colonies from single cells may not grow as well with no cells nearby (Pastrana et al., 2011). However, we have specifically demonstrated that plating at low densities ensures that most spheres are clonal (Coles-Takabe et al., 2008) and that the sphere assays are highly reproducible (Reynolds and Weiss, 1992; Tropepe et al., 2000; Imura et al., 2003; Pastrana et al., 2011; Vafaeva et al., 2024).

We had tried to isolate pancreatic sphere-forming cells from mouse E8.5, but we were unable to. However, we were able to isolate pancreatic sphere-forming cells from the hESC-derived PE (E8.5) stage, which then passaged into secondary spheres (Supplementary Figure 2B). The discrepancy between the ability to isolate insulin + spheres from the hESC-derived PE stage but not at the mouse-derived PE stage could be due to the species differences or because not all clonal pancreatic spheres are initiated by a self-renewing stem cell, but also can be initiated by progenitors that show limited self-renewal abilities. The inability to isolate spheres from the mouse E8.5 PE stage could indicate 1) technical issues, 2) that they do not exist at this stage (in mice), or 3) that they do emerge as early as E8.5 PE, but as a progenitor cell that does not acquire sphere-forming capability or stem cell qualities until the later E10.5 PP stage.

Species differences may likely account for our data with regards to the timeline in which pancreatic stem cells appear during embryonic development. A very recent study by Andersson-Rolf et al. (2024), created a pancreatic organoid from cells from trimesters 1 and 2 of the fetal human pancreas; this organoid recapitulates the fetal human pancreas organ. They have concluded that these organoids grow from a highly proliferative pancreatic stem cell and that this stem cell is fully multipotential, giving rise to all three pancreatic lineages: endocrine, exocrine, and duct (Andersson-Rolf et al., 2024). These results contrast mouse-derived pancreatic organoids, where cells are primarily fated toward ductal cells (Andersson-Rolf et al., 2024). Similar to what our study suggests, this study indicates that a pancreatic stem cell exists during human pancreatic development and that the stem cell’s contribution to the process of pancreatic development could be different in humans compared to mice.

With regards to the *Pdx1*-Cre; Rosa YFP reporter line used in our studies, there could be a concern because we have obtained a few YFP + neurons that differentiate, and one could interpret this as off-target labelling (Magnuson and Osipovich, 2013). However, we have shown previously that the developing pancreas contains both endoderm PDX1-derived precursors as well as neural crest-derived precursors. The neural crest cells migrate throughout the developing embryo, reach the developing gut at E9.5 in mice and begin to form the enteric nervous system (Durbec et al., 1996; Brokhman et al., 2019). Through using *Pdx1*-Cre, Rosa YFP lineage tracing mouse Arntfield and van derKooy et al. (2013) showed that the endoderm PDX1 precursors create the traditional clonal adult PMPs and all of their descendants (endocrine cells, exocrine cells and the small numbers of neurons) and that the Wnt-Cre; Rosa YFP lineage tracing mice, that marks the neural crest, gave rise to only neural crest stem cells which then lead to producing neurons in the intestine and some in the pancreas, but these two populations (Pdx1 and Wnt1 lineages) are distinct. Further, they showed that Wnt1-Cre lineage YFP expression is restricted to the NC derivatives at the adult time point. The same study showed that sections of Pdx1-Cre; ROSA-YFP pancreas showed YFP expression in expected pancreatic islet cell types, and sections from Wnt1-Cre; ROSA-YFP pancreas showed YFP expression in the neuronal and glial cells surrounding the islets demonstrating faithful on-target expression and minimal off-target YFP expression (Arntfield and van der Kooy, 2013). Our study also confirmed good on-target labelling through staining of the whole embryonic pancreas using anti-GFP (data not shown). Together, this supports the finding there is minimal off-target labelling of YFP expression and that pancreatic precursors that give rise to beta cells can be isolated at E11 (Figure 2). One explanation for the E11 pancreatic sphere from *Pdx1*-Cre; Rosa YFP mouse giving rise to a small amount of neurons (Figures 2C–F) is that the PDX1 lineage indeed gives rise to a few neurons. It has been previously shown, *in vivo*, that a small amount of PDX-1 progenitors migrate from the pancreas to the gut and, once there, differentiate into neurons to contribute a minor role to the enteric nervous system, supporting the idea that PDX1-derived neurons may have a biological function in the right environment (Brokhman et al., 2019).

In conclusion, although the results are preliminary, the isolation of pancreatic stem cells that demonstrate the ability to differentiate into both monohormonal insulin+ and glucagon + cells is possible from mouse age E11 (PP stage), and those isolated from later gestational periods such as E16 show self-renew. Further, polyhormonal pancreatic precursors were isolated at the hESC PE stage, and they ultimately became monohormonal insulin + cells in PE stage media. Isolation of hESC-derived pancreatic stem cells from possible contaminate cells during cytokine-directed differentiation and then exposing them to previously published beta-cell stage media may shorten the time to increase beta-cell purity. It is important to understand the ontogeny of beta-cells and hESC-derived pancreatic stem cells so that pancreatic precursors or stem cells can be identified and purified. Further successful characterization of hESC-derived pancreatic progenitors from the PE and especially the PP stages in terms of self-renewal ability and their ability to create monohormonal beta cells would optimally lead to an investigation on whether these progenitors could be pushed directly into a pure population of beta cells *in vitro* at that stage. Furthermore, this investigation would lead us to determine if these cells then create positive functional outcomes after transplantation. This would shorten the time to generate mature beta-cells compared to the time it takes to do this with current protocols involving hESC- beta-cell differentiation, which can take a month to accomplish. This characterization of human pancreatic stem cells from hESCs may allow the more rapid generation of the large numbers of beta cells necessary to meet the needs of accessible clinical transplantation to people who suffer from Type 1 or Type 2 diabetes.

## Data Availability

The raw data supporting the conclusions of this article will be made available by the authors, without undue reservation.
