# Supplementary material for: Pancreatic stem cells originate during the pancreatic progenitor developmental stage
Source: Front Cell Dev Biol. 2025 Feb 18;13:1521411. doi: 10.3389/fcell.2025.1521411 (PMC11876382; doi:10.3389/fcell.2025.1521411)
Supplement: Supplementary file 1 [file DataSheet1.pdf]

Supplemental Figure 1

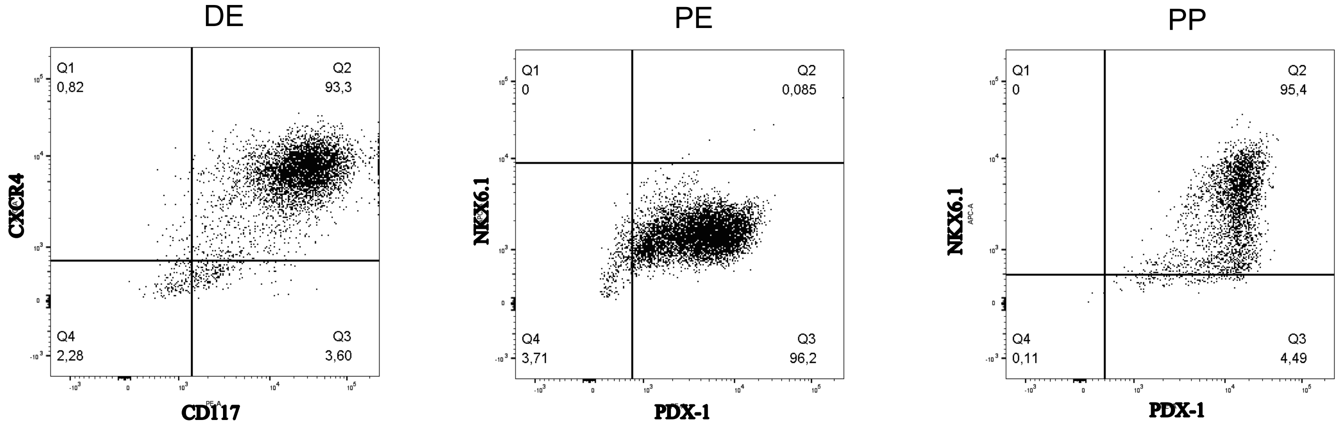

Figure S1. ins-GFP hESC differentiation successfully progresses towards pancreatic progenitor stage

hESC to beta cell cytokine differentiation was performed until the pancreatic progenitor stage according to previously described protocol (Day 0: Glutamine, CHIR 99021, Activin A, diluted monothiol glycerol (MTG) in RPMI; Day 3/Definitive Endoderm: Glutamine, bFGF, Activin A, Ascorbic acid in RPMI; Day 6/Posterior Foregut: Glutamine, B27, Dorsomorphin, FGF10, diluted MTG in RPMI; Day 8/Pancreatic Endoderm: Glutamine, Ascorbic acid, B27, FGF10, Noggin, Retinoic acid, SANT-1 in high glucose media; Day 13/Pancreatic Progenitor: Glutamine, B27, EGF, Noggin, Nicotinamide (NA) in high glucose media)[8]. Flow-cytometric analysis of major hESC pancreatic developmental stages that have distinct markers show that over 90% of definitive endoderm (DE) cells are double positive for CXCR4 and CD117, over 90% of pancreatic endoderm (PE) markers are singly positive for PDX1+, and over 90% of pancreatic progenitor cells (PP) are doubly positive for both PDX1+NKX6.1+.

Supplemental Figure 2

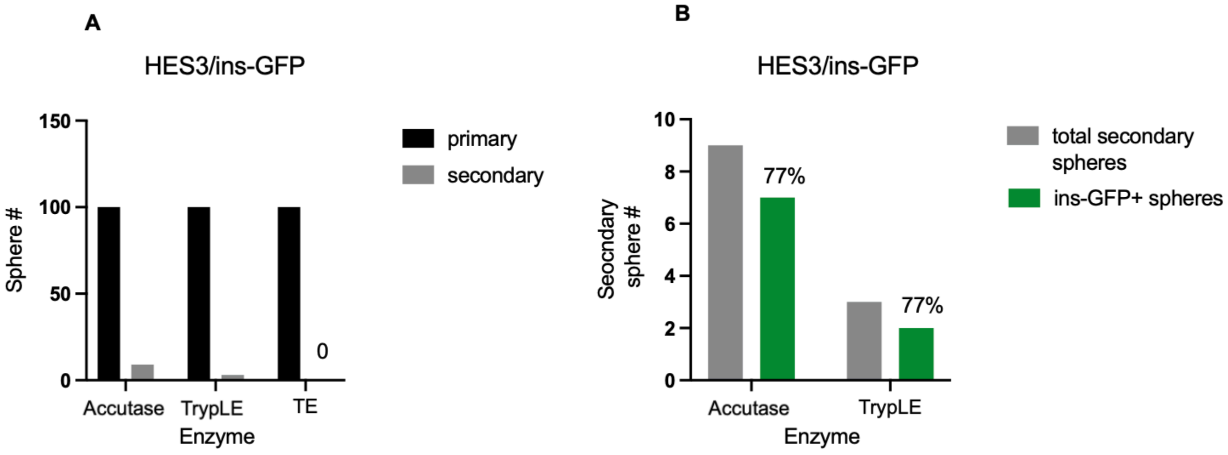

Figure S2. hESC-derived PE generated spheres passage in Accutase enzyme

(A) Bulk passaging of both ins-GFP+ and ins-GFP- primary spheres in three different enzymes, Accutase, TryplE and trypsin EDTA shows the cell survival after sphere dissociation iour s most optimal when done with Accutase compared to TryplE or trypsin EDTA, as indicated by number of secondary spheres generated from each condition. N=1 biological replicate, 100 spheres per condition.

(B) Bulk passaging of both ins-GFP+ and ins-GFP- primary spheres in the two best enzymes for survival (Accutase and TryplE) generate secondary spheres in which 77% of them are ins-GFP+ suggesting the potential passagability of primary PE spheres.

Supplemental Figure 3

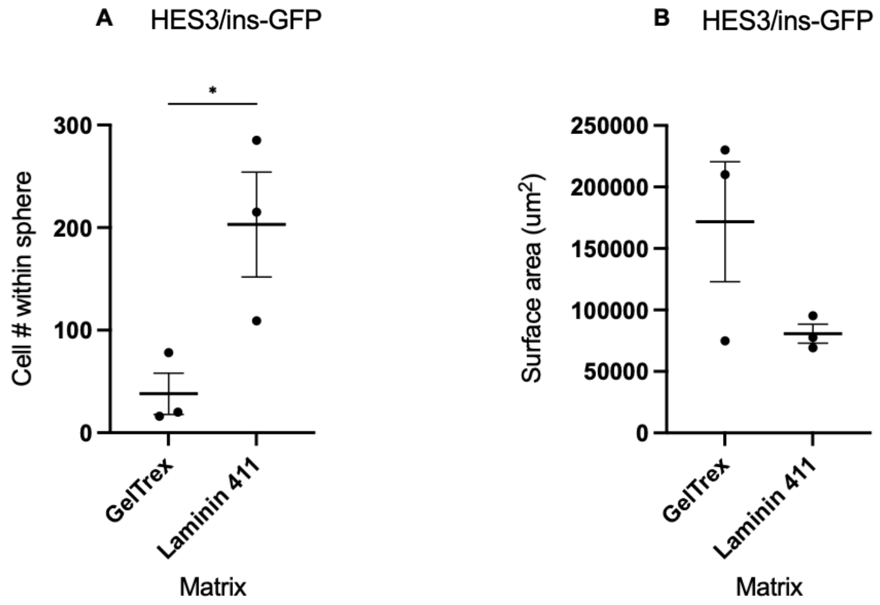

Figure S3. Laminin 411 extracellular matrix promotes survival of more cells within ins-GFP+ hESC derived PE spheres compared to that of Geltrex Similar sized (100-120µm) ins-GFP hESC-derived spheres generated from the PE stage were clonally plated onto either Laminin 411 or Geltrex and incubated in PE stage media for seven days. **(A)** Quantification of the number of cells that survived after 7 days within each sphere revealed that Laminin 411 promotes survival of the overall sphere progenitors, t(4)=3.003, p=0.0398; N=3 technical replicates. **(B)** Quantification of surface area (μm^2) of cell spread after 7 days revealed that Laminin 411 inhibits cell spread of the overall sphere progenitors, t(4)=1.845, p=0.1389; N=3 technical replicates.
